# Home Environment and Early Development of Rural Children: Evidence from Guizhou Province in China

**DOI:** 10.3390/ijerph18116121

**Published:** 2021-06-06

**Authors:** Lena Kuhn, Chengfang Liu, Tianyi Wang, Renfu Luo

**Affiliations:** 1 Leibniz Institute of Agricultural Development in Transition Economics, 06120 Halle, Germany; Kuhn@iamo.de; 2 China Center for Agricultural Policy, School of Advanced Agricultural Sciences, Peking University, Beijing 100871, China; cfliu.ccap@pku.edu.cn (C.L.); tianyi_ccap@pku.edu.cn (T.W.)

**Keywords:** home environment, learning material, early child development, gender gap, rural China

## Abstract

Delays in early child development are among the aspects underlying the persistent developmental gaps between regions and social strata. This study seeks to examine the relationship between the home environment and early child development in less-developed rural areas by drawing on data from 445 children from villages in Guizhou province in southwest China. A demographic questionnaire, the Home Observation Measurement of the Environment (HOME), and the Bayley Scales of Infant Development, version III (BSID-III), were used to measure the child’s demographic characteristics, home environment, and early development outcomes, respectively. Our data show that the sample children suffer a delay in various dimensions of child development and a deficit in the HOME scale. The results from a hierarchical regression model suggest that the availability of learning material at home, caregivers’ responsiveness and organization sub-scales are significantly positively correlated with the early development of sample children, after controlling for general socioeconomic status, health, and nutrition, and this correlation differs by gender. These results imply that the provision of learning material to households, promoting caregivers’ responsiveness and organization in less-developed rural areas could improve early child development among deprived children.

## 1. Introduction

An individual’s well-being is strongly related to his or her development between 0 and 8 years of age. Key early child development dimensions include physical, cognitive, motor, and social–emotional development [1]. Delays in early child development may contribute to the persistent developmental gaps between social strata or regions, and may even counteract all efforts to avoid the emergence of a middle-income trap [2].

Worldwide, many children are subject to various deprivations that fundamentally hinder their developmental progress. As estimated by a study in The Lancet, deprivation is so profound that nearly half (43%) of children in low- and middle-income countries may not achieve their full development potential [3]. A recent study discovered that 85% of children in rural China suffer from at least one kind of developmental delay [4].

Given its importance, a natural question emerges regarding what contributes to early child development. Empirical research has provided ample evidence with regard to the crucial impact of households’ socioeconomic status (SES) on child development [5]. Lugo-Gil et al. [6] provide evidence of how family resources contribute to children’s cognitive performance. Important pathways, such as the impact of nutrition on physiological and cognitive development, have been documented by numerous studies [7,8,9,10,11].

Many recent studies have examined the importance of the home environment in early child development. The home environment is defined as a safe and well-organized physical environment, with opportunities to play and explore, in which learning objects, toys, and books are supplied [12]. The WHO points out that the first three years of life are crucially influenced by the home environment [13]. This assertion has been confirmed by a growing number of empirical studies [14,15,16,17]. Specifically, some studies have found a positive effect of a stimulating home environment on children’s physical health [18], cognitive development [19], and linguistic ability [20]. In contrast, an unstimulating home environment, which is often found in low-income households, has been rated as one of the fundamental development risks for children under five years old [21].

Informed by these findings, it has been argued that improving the home environment can crucially promote child development, particularly in the early years [5]. Moreover, it has been noted that the home environment is not limited to physical infrastructure. Parenting practices constitute an equally, if not more, important component of the home environment. Some scholars argue that parenting practices, defined as context-specific behaviors of caregivers, can have a mediating effect between SES and child development [22,23]. Globally, parenting and education support has been found to be effective in improving children’s cognitive and psychosocial development, particularly in deprived regions or households [24]. For example, Gottfried et al. [25] observed a relationship between reading to children and children’s later reading achievement, rather than between the availability of reading material and reading outcomes. Bornstein et al. [26] documented a positive impact of maternal responsiveness on children’s cognitive development. The mediating effect of poor parenting on the negative relationship between transitory and persistent poverty was prominently highlighted by McLoyd [27].

Unfortunately, detailed and valid evidence on the role of the home environment in early childhood development among rural Chinese households is scarce. The majority of existing studies focus on parenting styles [28] or the difference in parenting styles between mothers and fathers in urban China [29,30]. As far as we know, only a few existing studies in the context of rural China focus on parenting traditions, patriarchal lifestyles, and related parenting styles [31].

To date, even fewer studies have explored the role of parenting practices in early child development in the context of China. Using data on migrant children from urban China, Liu et al. [32] found a significant positive association between lenient education styles and emotional development. Nyland et al. [33] describe formal childcare choices among urban Chinese families. One study linked the cognitive development delays among rural children to the absence of modern parenting among rural families [34]. Another study found child development delays were connected to passive parenting, expressed by a lack of interaction and play [35]. However, a comprehensive analysis of the nexus of child development and the home environment in China is lacking so far. Only one recent study examined the connection between child development and family care, and found serious developmental delays among rural Chinese children and a general lack of stimulating home environments [36]. It should be noted, however, that those authors’ assessment of a home environment was focused on learning materials and few play activities.

This study aims to close this research gap by providing novel evidence of the connection between various dimensions of the home environment and child development. In doing so, we draw on data from a unique comprehensive survey of the home environment and early child development among 445 children in underdeveloped rural areas of China. To assess their home environments, we employed the Home Observation Measurement of the Environment (HOME), which is based on observations by trained enumerators and reports by caregivers in six dimensions of the home environment. To measure child development, we used the Bayley Scales of Infant and Toddler Development, version III (BSID-III). In terms of socioeconomic and health/nutrition factors, we drew on information collected by an accompanying questionnaire conducted by trained enumerators with primary caregivers.

The rest of the paper is organized as follows: Section 2 introduces the methods of the study, including participants, procedures, measures, and the empirical approach. Section 3 presents the results. Section 4 discusses our main results, and Section 5 concludes the paper.

## 2. Materials and Methods

### 2.1. Participants

Our study builds on a dataset collected in Guizhou, one of the provinces with the lowest average income in China. The sample township was randomly selected from the list of all townships (excluding the township where the county seat is located) in a sample county in the sample prefecture, which is one of the poorest prefectures in the province. The sample township has 41 rural settlements in 9 villages, all of which were eligible to participate in the study. In total, 445 children, aged 4–26 months, and their caregivers participated in the study. While children were assessed for the BSID-III, their caregivers answered a general questionnaire, which included 39 items used for the HOME scores.

The Peking University Institutional Review Board (PU IRB), Beijing, China, approved the ethical assessment of the study (No. IRB00001052-17056). The purpose of the study was explained to the parents or guardians of all children, and verbal informed consent was obtained.

### 2.2. Procedures

The data we used in this study were collected from sample households over a three-week period in May 2017. A list of all registered births in each village was obtained from the local health officials. All children in the targeted age range in the sample villages were enrolled in the study. Teams of trained enumerators collected socioeconomic information from each sample household to measure the characteristics of the child, the caregiver, and the household. For collection of data pertaining to children, we recorded his/her gender, siblings, birth height, and weight. The exact age of each child was obtained from his or her birth certificate. At the caregiver level, we recorded his/her age and education level, as well as his/her relationship with the child. At the household level, we collected information about their socioeconomic status. Moreover, our survey also included a series of questions on the household’s parenting environment, in particular the parenting behavior toward the child.

### 2.3. Measures

#### 2.3.1. Child Development

We employed the BSID-III to measure child development. This test is a well-recognized and established tool in the psychological literature to diagnose certain developmental disorders [37]. The BSID-III divides the original Mental Development Index of earlier versions of the BSID into cognitive and language scales [38,39]. The cognitive scale assesses play skills, information processing (attention to novelty, habituation, memory, and problem-solving), counting, and number skills. The language scale consists of two sub-scales, namely expressive and receptive communication, which measure communication skills such as language and gestures. The motor scale evaluates skills associated with eye movements, perceptual–motor integration, motor planning, motor speed, and movements of the limbs and torso, distinguishing fine motor and gross motor skills. The social–emotional scale measures emotional and social functioning as well as sensory processing [38].

To assess the four sub-scales, the test measures a child’s performance on a series of tasks using a standardized toy kit. For each passed task or item, one point adds to the total raw score in the respective dimension. Overall, the cognitive sub-scale consists of 91 items, the language sub-scale of 97 items, the motoric sub-scale of 138 items, and the social–emotional sub-scale of up to 35 items. Raw scores are converted into scaled scores by considering the developmental progress according to child age and composite scores, which allows for a comparison between scales.

The test was administered one-on-one using a set of standardized toys and a detailed scoring sheet. All enumerators attended a weeklong training course on how to administer the BSID-III in the field.

While many studies typically use one of these norm-referenced scores (scaled scores or composite scores), we followed Attanasio et al. [40] or Sylvia et al. [41] and conducted the analysis with raw scores, except for some comparative analysis in Section 3.1. This is because, as far as we know, no child development norm is available for the Chinese population yet. Norm-referenced scores are only employed for descriptive comparisons between dimensions and with other international samples.

#### 2.3.2. Home Environment

We employed HOME to assess the quality of a child’s family environment. The infant and toddler version of HOME includes six dimensions: (a) responsiveness; (b) acceptance (lack of punitive action); (c) organization; (d) play/learning material; (e) involvement (stimulation of development); and (f) variety of experience [42,43]. The scores for each of these items are calculated as a sum of binary questions, which are answered either by primary caregivers (usually the mother) or enumerators based on their observations of the home environment or parent–child interaction. The six sub-scores are used to generate the overall score. The detailed questions are listed in the Appendix A.

To achieve our research objectives, we needed to differentiate between aspects of the home environment. Broadly, we focused on two aspects: aspects that are mostly driven by income (for instance, play material), and aspects related to non-material parenting styles (such as responsiveness or acceptance). To this end, we introduced not only the aggregated home scores, but also the underlying sub-scores.

For this study, we used a slightly shortened version of HOME to adapt to the local context. According to Bradley and Corwyn [43], such adaptations are common practice when employing HOME scales in different cultural backgrounds. Direct questions to the caregiver were posed in a low-key, semi-structured interview style to avoid interview bias and minimize stress for respondents. Some of the questions were supported with an illustration (for instance, of toys) to avoid misunderstandings on the part of the caregiver.

#### 2.3.3. Analytical Plan

To estimate the association between the home environment and child development, we employed a hierarchical multiple regression model [44]. This model allowed us to test whether or not a specific set of independent variables explained a statistically significant amount of variance in a dependent variable after accounting for all other variables. In the context of child development, hierarchical regression models were used, for instance, by Hoff [45] or Kim et al. [46]. We also controlled for village-level fixed effects and clustered standard errors at the natural village-level.

Variables are entered in sequence of their causal priority and presumed theoretical importance in the hierarchical multiple regression model [47]. In the first step, we introduced a block of socioeconomic variables to control for family characteristics. A block of nutrition and health variables were entered in the second step. Home environment variables, the dimension of main interest, were finally added to the hierarchical multiple regression model.

Following the literature, we controlled for the following socioeconomic variables. Specifically, we controlled for low-income levels (<10,000 RMB annual income) as a proxy for a poor economic environment. To control for potential cultural effects, we included a binary variable for ethnic minorities. Due to the potential interaction of parents’ education and parenting as well as child development [48], we also introduced parental education levels as a control variable. To avoid endogeneity, we only introduced the education level of the parent that was not the main caregiver. Where neither the father nor the mother was the main caregiver, we used the average education level of both parents. Furthermore, we included the age of the child to make up for the lack of country-specific age-standardized development scores, as well as the gender of the child to control for potential differences in parenting styles between the genders [49]. Finally, we introduced the number of siblings to control for cases in which the child had to share parents’ attention and resources with other children in the household [50].

We also took into account the effects of nutrition and health impacts [7,8,9,10,11]. Specifically, we introduced a binary variable for the intake of nutrition supplements by mothers during pregnancy and underweight of children at birth (defined as a birthweight of less than 2500 g), both controlling for physiological development delays at birth. Furthermore, we controlled with a binary variable for smoking parents, which might affect prenatal and postnatal physiological development. Feeding habits were captured by a binary variable determining whether mothers ever breastfed their child.

## 3. Results

### 3.1. Descriptive Statistics

Table 1 presents descriptive statistics on the sample children and family. The final sample size for the study is 445 children and their caregivers. The average age of children in the study was 14.6 months, ranging from 4 months to 26 months. More than half (58%) of the sample children were boys, which is slightly higher than the gender ratio at age 0–4 years reported in the provincial census (56%) [51]. In total, 23% of the sample children belonged to an ethnic minority group. In terms of household socioeconomic status, 19% of the sample children belonged to low-income families with an annual gross income of 10,000 RMB or less. In more than two-thirds (68%) of cases, the mother was the primary caregiver and the mean education level of the parents was less than 8 years. On average, the sample children had 1.3 siblings (SD = 1.17), and 26.3% were an only child. The average birthweight of sample children was 3226 g (SD = 670.22), with 7% of them weighing below 2500 g and thus considered of low birthweight. Around 80% of the children had been breastfed for at least some time during infancy, and 44% had received nutritional supplements (calcium or iron) at some point. About 60% of the sample children experienced environmental tobacco smoke exposure.

Table 2 presents summary of statistics on child development as measured with BSID-III. To allow for a comparison between our results and those found in the literature, we report both composite scores and raw scores. As for the index design, the possible composite scores of the cognitive and social–emotional sub-scales range between 55 and 145. The possible composite scores of the language sub-scale range between 47 and 153, while the composite scores of the motoric sub-scale range between 46 and 154. In our sample, the composite scores for cognitive, language, motoric, and social–emotional development lay between 46 and 145, with standard deviations between 11.9 and 16.3 (see Table 3). We found the lowest average score for social–emotional development (M = 85.35, SD = 11.91) and the highest for motoric development (M = 95.35, SD = 15.78). Searching for healthy population means suitable for China, we note Wang et al. [4], who use benchmark values of 105 for the cognitive scale, 109 for the language scale, 107 for the motor scale, and 100 for the social–emotional scale. The sample means reach only 82–89% of these benchmarks of healthy population means, indicating that the sample population lagged behind health populations by 11–18%. These results are in line with previous assessments of low levels of child development among rural Chinese children by Wang et al. [4] and Yue et al. [34]. The mean value of the raw scores for cognitive, receptive communication, expressive communication, fine motor, gross motor, and social–emotional development are 43.56, 15.24, 16.01, 30.49, 42.19, and 73.20, respectively.

**Figure 1:**
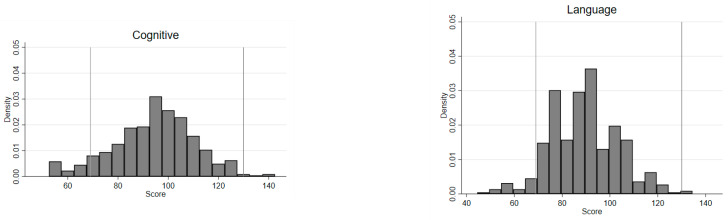

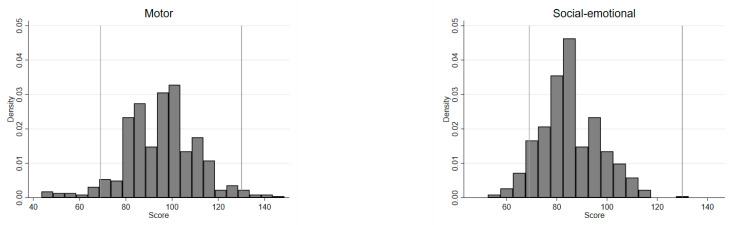
Density distribution of BSID-III composite scores.

To assess the overall performance across dimensions, Figure 1 displays the density distributions of BSID-III composite scores. The 2nd and 98th percentiles mark the thresholds for ‘extremely low’ and ‘very superior’ scores, respectively, in any of the four composite BSID-III scales [38]. As illustrated in Figure 1, the means of all four development scales were closer to the lower threshold (score = 69) than to the upper threshold (score = 130). While 4–6% of the sample scored ‘extremely low’, only up to 2% of the sample achieved ‘very superior’ scores in the respective scales.

**Figure 2:**
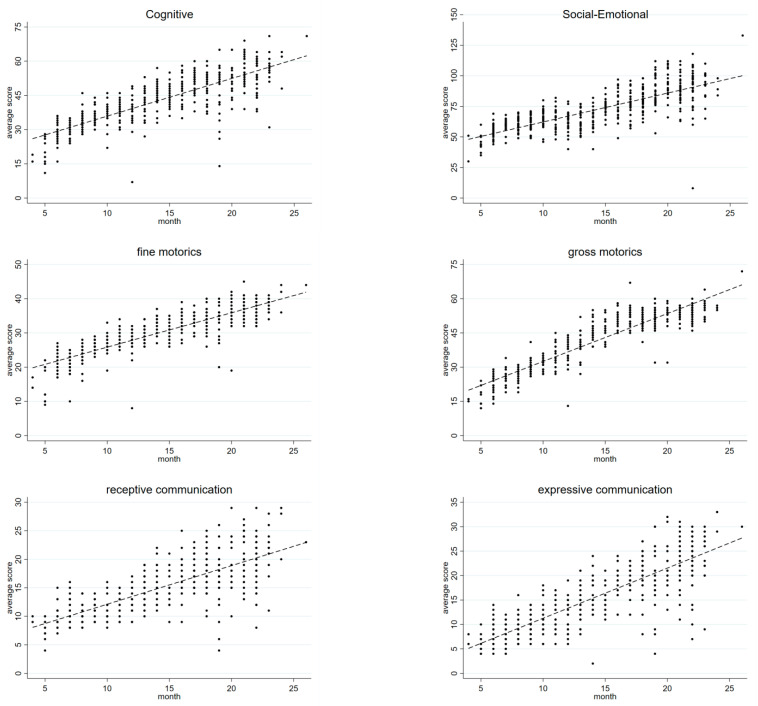
BSID-III raw scores by age.

As expected, the development of raw scores increased with the age of children (Figure 2). We observed a rapid increase in scores between the ages of 4 and 26 months, particularly for gross motor (365%), expressive communication (329%), and cognitive scales (306%), whereas social–emotional (228%) and receptive communication (142%) increased at lower rates.

Another distinctive feature is the cognitive development disparity between genders. In our sample, the cognitive raw score of boys (M = 44.82, SD = 11.13) was slightly higher than their female peer group’s raw score (M = 41.85, SD = 11.04). The difference between the genders in terms of cognitive development was statistically significant (*p* < 0.01). At the same time, we found a statistically significant difference between the genders in terms of gross (*p* = 0.021) and fine motor development (*p* = 0.024). Gender differences in terms of language and social–emotional raw scores were not statistically significant (see Table 3). For other demographic features like ethnicity, no significant group differences in BSID-III raw scores could be found.

Our results show that the home environment varies considerably between the six HOME sub-scales (Table 4). For the responsiveness, acceptance, and involvement sub-scale, the mean score of sample households was closest to the maximum possible scores of 10, 7, and 6, respectively. In terms of the organization, learning material, and variety sub-scale, the mean scores of sample households were only half or less than half of the maximum possible scores of 5, 7, and 4, respectively. Overall, no household scored the theoretically maximum achievable score, and no household scored less than 10 overall. Thus, the total score varied between 25 and 64% of the maximum possible points.

To put these results into perspective, a comparison with other datasets is necessary. As a benchmark for a healthy sample population, we chose the study of Bradley et al. [42], which is based on the NICHD Study of Early Child Care among over 1300 children in the United States of America. In the Appendix, we compare the pass rate of both samples, which is defined as the rate of positive answers to the binary item questions or observations contributing to the HOME scores. Overall, the pass rate of our sample for learning material is 41 percentage points (p.p.) lower than that of the US sample collected by Bradley et al. Furthermore, we also found lower pass rates in the Chinese sample for organization (by 17 p.p.), variety (by 35 p.p.), responsiveness (by 11 p.p.), and acceptance (by 3 p.p.). Meanwhile, in some items that reflect emotional attention to the child (kisses or talking to the child), pass rates are considerably higher in the Chinese sample than in the US sample collected by Bradley et al. As illustrated by Bradley et al. [43], cultural differences make cross-country comparisons of HOME scores difficult. Nevertheless, the distinct differences in some of the HOME sub-scales are striking.

**Figure 3:**
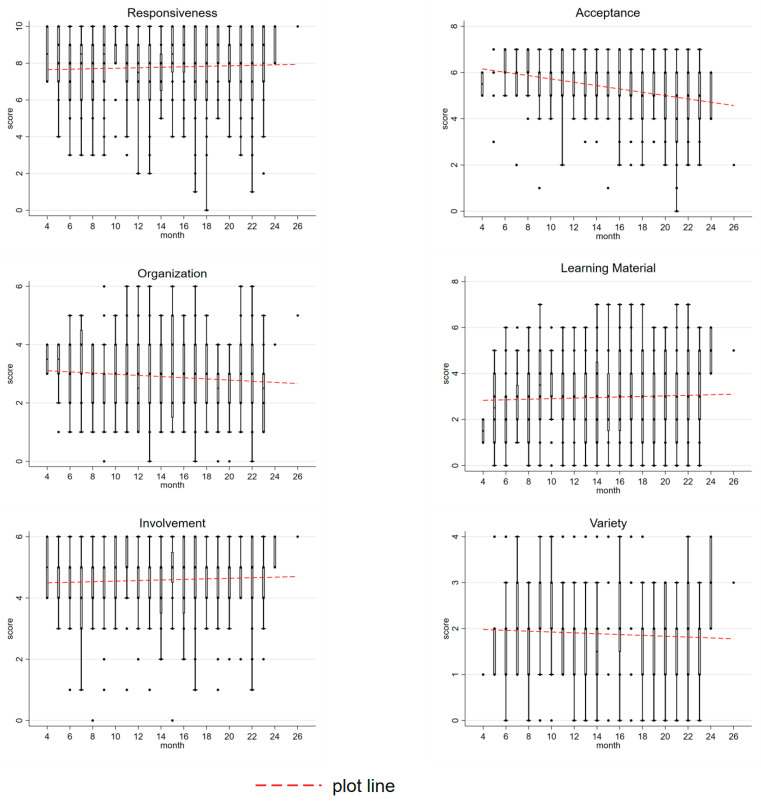
Boxplots for HOME scores by age.

To trace the development of the home environment across child age, Figure 3 displays boxplots of HOME scores across age groups, as well as a plotline. Some sub-scales of the HOME feature a slight upward trend as children’s ages increase, for instance, the responsiveness, involvement, and learning material sub-scales. This trend is likely a response to the increase in activity and needs of children with age. For some other sub-scales, we found a slight downward trend with age, namely with regard to home organization and variety. Moreover, there is a noticeable dramatic downward trend for the acceptance sub-scale, which might be either a reaction to children’s increased activity and/or a decreased tolerance for perceived negative behavior on the part of caregivers.

When testing for disparities between boys and girls, we found significant gender differences only in terms of learning material. The learning material sub-score in households with a male child is, on average, 0.4 score points higher than for households with a female child, a finding that might explain the gender differences in some development scores. Between Han and ethnic minorities, we found significant differences only for the acceptance sub-score, where non-Han households feature a slightly higher score (5.6) than Han households (5.3) (Table 5).

### 3.2. Regression Results

Table 6 presents the results of the hierarchical regression analysis of six disaggregated development raw scores on several potentially related household and child characteristics as well as the HOME score. The variables entered the regression in three groups—general socioeconomic characteristics, nutrition, and health-related characteristics—and the HOME score, taking into account previous steps but not subsequent steps.

Our regression results show that the HOME score is statistically significantly related to children’s language (*p* < 0.001), gross motor (*p* < 0.05), and social–emotional development (*p* < 0.001). For each additional point increase in the HOME score, the receptive communication, expressive communication, gross motor, and social–emotional sub-scores (raw scores) increased by 0.10, 0.17, 0.11, and 0.23 points, respectively. The contribution of HOME score to the total model’s prediction power (measured by ΔR^2^), however, was small, from 0.001 for the cognitive score to 0.014 for expressive communication.

A stronger effect was found for socioeconomic, nutrition, and health-related variables. As expected, an increase in age of one month at the mean translated into a significant increase in all development scores, with a monthly average growth rate of between 0.70 (receptive communication) and 2.41 (social–emotional). However, being in the lowest income class was statistically significantly related to a 2.63-point lower cognitive raw score (*p* < 0.001) and a 0.96-point lower receptive communication raw score (*p* < 0.05). This significant correlation indicates the importance of material welfare for parenting quality. Furthermore, we found that, with each additional sibling, both the cognitive raw score and the expressive communication raw score decreased by 0.63 points and 0.56 points, respectively. This finding reflects the ambivalent role of siblings in early child development, where potential positive effects on cognitive and language development can be outweighed by the need to split attention and resources [50]. Cultural factors also seemed to be at play, significantly so in the language dimension. Children of non-Han parents featured a 0.89-point higher expressive communication raw score than their Han peer group. Furthermore, parents’ education seemed to influence child development; each additional educational level was related to higher cognitive, communication, and motor scores. When controlling for other factors, the gender of the child was not related to significant differences in child development. The lack of gender effects to certain degrees adds weight to the notion that greater parenting efforts for boys might have been the source of the slight development lead among male children.

Finally, we also found a certain contribution of health and nutrition variables. The intake of nutrition supplements during pregnancy was related to a 0.86-point increase in gross motor skills. A 10% increase in birth weight (in grams) was related to a 0.30-point increase in the cognitive raw score and a 0.20-point increase in fine motor raw score. Children that had been breastfed at some point in their infancy had a 1.13-point higher level of gross motor development on average. In smokers’ households, children had significantly lower receptive communication raw scores (0.59 points) and motor raw scores (0.61 points).

The second series of hierarchical regression models reveal further insights into the particular effects of home environment components. This time, the regression was conducted for the six HOME sub-scales separately: acceptance, responsiveness, involvement, organization, variety, and learning material.

The results of this more detailed analysis are displayed in Table 7, where we provide the results for disaggregated home environments. It should be noted that, since home environment variables enter the regression in the third and final step, the disaggregation does not affect the results for steps 0–2 given in Table 6. In the disaggregated models, we found a statistically significant correlation with child development for the responsiveness, organization, and learning material sub-scales, albeit organization was only significant at the 10% level. In the responsiveness sub-scale, a one-point increase led to 0.137- and 0.242-point increases in receptive and expressive communication, respectively. This positive correlation of parental responsiveness to child development is in line with many earlier studies [52]. A one-point increase in terms of organization was accompanied by a 0.279-point increase in expressive communication score and a 0.427-point increase in terms of gross motor score.

Among all HOME sub-scales, learning material had both the strongest and the most diverse connection with child development. A one-point increase in learning material was associated with considerable and statistically significant increases in terms of cognitive scores (0.525 points), expressive communication score (0.256 points), fine motor score (0.272 points), and gross motor score (0.399 points). Furthermore, a one-point increase in learning material was also related to an increase in receptive communication (0.201 points), though only at the 10% significance level. For the acceptance, involvement, and variety sub-scales, no significant connection with child development could be found.

## 4. Discussion

This study shows that the sample children exhibited a delay in various dimensions of child development, reaching only 82–89% of these benchmarks of healthy population means, indicating that the sample population lagged behind healthy populations by 11–18%. We also found that these delays were closely related to HOME scale. Compared with the international benchmark, our sample households showed considerably lower scores for learning materials, organization, variety, and responsiveness. While this finding certainly requires a comparison with samples from other countries, it does confirm patterns revealed by other studies, placing an emphasis on parenting, specifically parents’ involvement.

Previous studies have shown a moderate correlation between HOME scale and the cognitive and language development of children across all age cohorts, as summarized by Bradley [53]. However, our study sample only exhibited correlations between HOME scale, language, and social–emotional development. This difference might suggest that HOME scale and the parenting practices associated with it may, to a certain degree, reflect a household’s socioeconomic status [54] and thus act as mediating factors [53].

Many studies argued that there is evidence of a more specific influence when investigating the disaggregated effects of each HOME sub-scale [55,56]. This perspective is confirmed by our results, which reveal a significant and moderate relationship between responsiveness and language development, as well as a significant relationship between learning material and cognitive development, which did not manifest in the aggregated HOME score. This study also confirms a significant moderate association between a child’s motor development and the respective household’s learning material and organization.

However, the influence of HOME scales on social–emotional development could not be supported by the disaggregated data. A possible explanation for this finding may be the cultural particularities of the sample. Guizhou is one of the provinces with the highest share of ethnic minorities. As investigated by various studies (e.g., Bornstein [57] and Richman et al. [58]), there are considerable cultural differences in maternal responsiveness to infants, in particular concerning the extent and speed of tactile responsiveness and verbal methods of responsiveness. Furthermore, the sample consisted of a relatively large share of children whose main caregivers are not their parents, but their grandmothers. Those caregivers may have exhibited responsiveness to children during the interview, but are overwhelmed with farming and childcare tasks during everyday life [59]. The high-intensity care required for small infants is reported to have detrimental health effects on grandparents [60], especially for elderly caregivers who are not adequately supported financially by their migrant children [61].

## 5. Conclusions

In conclusion, this study shows that the sample children suffer a delay in cognition, language, motor, and social–emotional development and a deficit in the parenting environment according to the HOME scale and sub-scales. The results also demonstrate the existence of a positive correlation between HOME scale and children’s language, gross motor, and social–emotional development. Our findings further reveal a heterogeneous effect among HOME sub-scales and child development, in which the learning material, responsiveness, and organization sub-scales are positively associated with early child development. These results imply that parenting services on the provision of learning material, the improvement of caregivers’ responsiveness and organization in less-developed rural areas could contribute to early child development among deprived children and narrow the persistent gaps early child development.

## Figures and Tables

**Table 1:** Descriptive statistics of child and family characteristics.

Variable	Unit	Mean	SD	SE (mean)	Min	Max	N
Age	months	14.620	5.478	0.260	4	26	445
Gender	1 = male, 2 = female	1.425	0.495	0.023	1	2	445
Ethnic minority	0 = no, 1 = yes	0.225	0.418	0.020	0	1	445
Low-income group	0 = no, 1 = yes	0.193	0.395	0.019	0	1	445
Parent’s education	years	7.787	3.373	0.160	0	19	445
Siblings	number	1.328	1.166	0.055	0	7	445
Birthweight	grams	3226.169	670.224	31.807	1500	6500	444
Breastfeeding	0 = no, 1 = yes	0.791	0.407	0.019	0	1	444
Nutrition supplements	0 = no, 1 = yes	0.440	0.497	0.024	0	1	445
Parent smoking	0 = no, 1 = yes	0.607	0.489	0.023	0	1	445

**Table 2:** Descriptive statistics of BSID-III scores (*n* = 445).

Score Type	Scale	Mean	SD	Min	Max
BSID-III composite scores	Cognitive	94.45	16.32	55	140
	Language	89.68	13.83	47	132
	Motoric	95.35	15.78	46	145
	Social–emotional	85.35	11.91	55	130
BSID-III raw scores	Cognitive	43.56	11.17	7	71
	Receptive communication	15.24	4.91	4	29
	Expressive communication	16.01	6.87	2	33
	Fine motor	30.49	6.48	8	45
	Gross motor	42.19	12.63	12	72
	Social–emotional	73.20	17.01	8	133

**Table 3:** BSID-III raw scores by gender.

Scale	Male	Female	t-Statistics	*p*-Value
Cognitive	44.82	41.85	−2.79	0.006
Receptive communication	15.45	14.96	−1.04	0.298
Expressive communication	16.36	15.53	−1.27	0.206
Fine motor	31.08	29.68	−2.26	0.024
Gross motor	43.38	40.59	−2.31	0.021
Social–emotional	74.32	71.68	−1.62	0.105

**Table 4:** Descriptive statistics of HOME scores (*n* = 444).

Sub-Score	Mean	SD	Min	Max
Responsiveness	7.79	2.09	0	10
Acceptance	5.39	1.31	0	7
Organization	2.89	1.41	0	5
Learning material	2.96	1.88	0	7
Involvement	4.59	1.21	0	6
Variety	1.88	0.96	0	4
Total score	25.50	5.37	10	39

**Table 5:** HOME sub-scores by gender.

Sub-Score	Gender			Ethnicity		
	Male	Female	*p*-Value	Han	Non-Han	*p*-Value
Responsiveness	7.74	7.85	0.57	7.89	7.55	0.12
Acceptance	5.38	5.40	0.89	5.29	5.62	0.01
Organization	2.83	2.98	0.26	2.89	2.89	0.99
Learning material	3.12	2.74	0.03	2.97	2.94	0.89
Involvement	4.56	4.63	0.58	4.63	4.52	0.39
Variety	1.89	1.88	0.93	1.88	1.88	1.00

**Table 6:** Hierarchical regression results (HOME score).

	Dependent Variables	Cognitive	Language		Motor		Social–Emotional
Explanatory Variables			Receptive Communication	Expressive Communication	Fine Motor	Gross Motor	
Step 0: Village-Level Fixed Effects							
Step 1: Socioeconomic Variables							
Low-income		−2.627 ***	−0.962 **	−0.824	−0.725	−1.279 *	−0.174
		(0.787)	(0.364)	(0.527)	(0.461)	(0.649)	(1.721)
Ethnic minority		−0.313	0.357	0.894*	−0.511	−0.360	0.603
		(0.892)	(0.441)	(0.521)	(0.427)	(0.741)	(1.373)
Parent’s education		0.301 *	0.120 ***	0.142 **	0.105	0.230 **	0.298
		(0.153)	(0.062)	(0.070)	(0.075)	(0.098)	(0.230)
Age		1.671 ***	0.702 ***	1.050 ***	1.001 ***	2.111 ***	2.414 ***
		(0.052)	(0.034)	(0.048)	(0.033)	(0.054)	(0.092)
Male		1.079	−0.251	−0.263	0.262	0.500	−0.234
		(0.732)	(0.317)	(0.415)	(0.383)	(0.602)	(1.198)
Siblings		−0.630 **	−0.190	−0.557 ***	0.078	−0.263	−0.582
		(0.262)	(0.151)	(0.158)	(0.167)	(0.249)	(0.591)
Constant		15.370 ***	4.970	0.835	15.200	10.577	39.387
		(1.822)	(0.826)	(0.861)	(15.200)	(1.364)	(2.724)
R^2^		0.685	0.595	0.696	0.731	0.835	0.596
ΔR^2^		0.648	0.571	0.655	0.678	0.790	0.563
Step 2: Nutrition and health variables							
Nutrition supplements		0.439	0.309	0.336	0.041	0.864 *	1.172
		0.598	(0.289)	(0.346)	(0.383)	(0.497)	(1.053)
Birthweight		2.963 *	0.971	1.294	1.996 *	0.009	4.641
		1.473	(0.613)	(1.054)	(0.654)	(1.166)	(2.799)
Breastfeeding		0.597	0.651	0.273	0.663	1.126 *	0.429
		0.788	(0.408)	(0.388)	(0.498)	(0.620)	(1.411)
Smoke		−0.685	−0.586*	−0.437	−0.606 *	−0.343	−1.183
		0.674	(0.323)	(0.422)	(0.359)	(0.496)	(1.008)
Constant		−7.893	−2.849	−9.429	−0.744	9.807	2.617
		11.741	(4.966)	(8.479)	(5.412)	(9.588)	(2.617)
R^2^		0.703	0.610	0.706	0.739	0.838	0.605
ΔR^2^		0.018	0.015	0.010	0.008	0.002	0.010
Step 3: Home environment							
HOME		0.060	0.102 ***	0.172 ***	0.050	0.113 **	0.227 ***
		(0.058)	(0.034)	(0.038)	(0.039)	(0.043)	(0.079)
R^2^		0.703	0.620	0.720	0.741	0.839	0.609
ΔR^2^		0.001	0.010	0.014	0.001	0.002	0.004
Constant		−5.207	−2.704	−9.186	−0.674	9.962	2.937
		12.037	4.953	8.315	5.369	9.396	22.275
F-test		170.52	70.29	83.78	186.27	171.56	102.55
Number of observations		444	444	444	444	443	444

Note: Standard errors in parentheses, * *p* < 0.1; ** *p* < 0.05; *** *p* < 0.01.

**Table 7:** Hierarchical regression results (stage 3 with detailed HOME scores).

	Dependent Variables	Cognitive	Receptive Communication	Expressive Communication	Fine Motor	Gross Motor	Social–Emotional
Explanatory Variables							
Step 0: Village-level fixed effects							
Step 1: Socioeconomic variables							
Low-income		−2.627 ***	−0.962 **	−0.824	−0.725	−1.279 *	−0.174
		(0.787)	(0.364)	(0.527)	(0.461)	(0.649)	(1.721)
Ethnic minority		−0.313	0.357	0.894 *	−0.511	−0.360	0.603
		(0.892)	(0.441)	(0.521)	(0.427)	(0.741)	(1.373)
Parent’s education		0.301 *	0.120 ***	0.142 **	0.105	0.230**	0.298
		(0.153)	(0.062)	(0.070)	(0.075)	(0.098)	(0.230)
Age		1.671 ***	0.702 ***	1.050 ***	1.001 ***	2.111 ***	2.414 ***
		(0.052)	(0.034)	(0.048)	(0.033)	(0.054)	(0.092)
Male		1.079	−0.251	−0.263	0.262	0.500	−0.234
		(0.732)	(0.317)	(0.415)	(0.383)	(0.602)	(1.198)
Siblings		−0.630 **	−0.190	−0.557 ***	0.078	−0.263	−0.582
		(0.262)	(0.151)	(0.158)	(0.167)	(0.249)	(0.591)
Constant		15.370 ***	4.970	0.835	15.200	10.577	39.387
		(1.822)	(0.826)	(0.861)	(15.200)	(1.364)	(2.724)
R^2^		0.685	0.595	0.696	0.731	0.835	0.596
ΔR^2^		0.648	0.571	0.655	0.678	0.790	0.563
Step 2: Nutrition and health variables							
Nutrition supplements		0.439	0.309	0.336	0.041	0.864 *	1.172
		0.598	(0.289)	(0.346)	(0.383)	(0.497)	(1.053)
Birthweight		2.963 *	0.971	1.294	1.996 *	0.009	4.641
		1.473	(0.613)	(1.054)	(0.654)	(1.166)	(2.799)
Breastfeeding		0.597	0.651	0.273	0.663	1.126 *	0.429
		0.788	(0.408)	(0.388)	(0.498)	(0.620)	(1.411)
Smoke		−0.685	−0.586 *	−0.437	−0.606 *	−0.343	−1.183
		0.674	(0.323)	(0.422)	(0.359)	(0.496)	(1.008)
Constant		−7.893	−2.849	−9.429	−0.744	9.807	2.617
		11.741	(4.966)	(8.479)	(5.412)	(9.588)	(2.617)
R^2^		0.703	0.610	0.706	0.739	0.838	0.605
ΔR^2^		0.018	0.015	0.010	0.008	0.002	0.010
Step 3: Home environment							
Responsiveness		−0.226	0.137 *	0.242 ***	0.032	0.073	0.301
		(0.144)	(0.076)	(0.081)	(0.093)	(0.126)	(0.211)
Acceptance		0.138	−0.006	−0.115	0.003	−0.052	0.337
		(0.246)	(0.109)	(0.194)	(0.109)	(0.176)	(0.452)
Organization		0.128	0.224	0.279 *	0.064	0.427 *	−0.058
		(0.309)	(0.147)	(0.161)	(0.155)	(0.216)	(0.438)
Learning Material		0.525 ***	0.201 *	0.259 **	0. 272 **	0.399 **	0.252
		(0.190)	(0.118)	(0.123)	(0.107)	(0.189)	(0.321)
Involvement		−0.231	−0.155	−0.018	−0.178	−0.284	0.486
		(0.218)	(0.148)	(0.218)	(0.175)	(0.253)	(0.518)
Variety		0.019	0.063	0.129	−0.052	−0.241	−0.052
		(0.389)	(0.181)	(0.231)	(0.201)	(0.389)	(0.627)
Constant		−2.144	−1.862	−8.280	0.671	11.934	2.132
		(12.676)	(4.957)	(8.538)	(5.529)	(9.419)	(22.901)
F-test		223.325	73.204	100.777	188.628	209.902	144.680
Number of observations		444	444	444	444	443	444

Note: Standard errors in parentheses, * *p* < 0.1; ** *p* < 0.05; *** *p* < 0.01.

## Data Availability

The data presented in this study are available on request from the corresponding author.

## Appendix A

**Table A1:** Infant-Toddler Child Care HOME Inventory with pass rates.

	Item	NICHD	This Study
Responsiveness	1. Caregiver spontaneously vocalizes to child at least twice	97.9	77.0
	2. Caregiver responds verbally to child’s vocalizations or verbalizations	89.2	68.0
	3. Caregiver tells child name of object or person during visit	80.2	49.6
	4. Caregiver’s speech is distinct, clear, and audible	97.2	82.2
	5. Caregiver initiates verbal interchanges with visitor	95.4	79.5
	6. Caregiver converses freely and easily	98.5	88.5
	7. Caregiver permits child to engage in messy play	83.2	-
	8. Caregiver spontaneously praises child at least twice	64.7	60.1
	9. Caregiver’s voice conveys positive feelings toward child	95.3	92.3
	10. Caregiver caresses or kisses child at least once	80.7	93.9
	11. Caregiver responds positively to praise of child offered by visitor	94.8	87.4
	Average	88.8	77.9
Acceptance	12. Caregiver does not shout at child	93.0	89.4
	13. Caregiver does not express overt annoyance with or hostility to child	90.7	98.0
	14. Caregiver neither slaps or spanks child during visit	97.4	93.7
	15. No more than one instance of physical punishment during past week	81.4	68.0
	16. Caregiver does not scold or criticize child during visit	87.4	86.9
	17. Caregiver does not interfere with or restrict child three times during visit	55.9	62.4
	18. At least 10 books are present and visible	53.9	40.8
	Average	80.0	77.0
Organization	19. Caregiver is one of no more than three regular substitutes used for child	93.0	67.3
	20. Child is taken on an outing at least once a week	57.7	53.4
	21. Child gets out of house at least four times a week	84.8	56.8
	22. Caregiver has an emergency medical and/or accident plan	69.6	-
	23. Child has a special place for toys and treasures	81.7	39.9
	24. Child’s play environment is safe	41.0	71.5
	Average	71.3	57.8
Learning Materials	25. Muscle activity toys or equipment	87.8	39.4
	26. Push or pull toy	80.1	34.2
	27. Stroller or walker, kiddie car, scooter, or tricycle	87.4	74.8
	28. Caregiver provides toys for child to play with during the visit	97.7	37.8
	29. Cuddly toy or role-playing toys	96.9	44.4
	30. Learning facilitators—mobile, table and chair, high chair, playpen	75.8	-
	31. Simple eye–hand coordination toys	93.3	48.4
	32. Complex eye–hand coordination toys	62.9	16.9
	33. Toys for literature and music	71.7	-
	Average	83.7	42.3
Involvement	34. Caregiver keeps child in visual range, looks at often	81.4	91.2
	35. Caregiver talks to child while doing household work	60.3	89.2
	36. Caregiver consciously encourages developmental advance	81.2	89.9
	37. Caregiver invests maturing toys with value via personal attention	65.2	79.1
	38. Caregiver structures child’s play periods	68.6	81.8
	39. Caregiver provides toys that challenge child to develop new skills	65.2	28.2
	Average	70.3	76.6
Variety	40. Caregiver reads stories to child at least three times weekly	74.5	16.2
	41. Child eats at least one meal with caregiver and/or other children	93.0	77.7
	42. Caregiver and child visit or receive from neighbors or friends about once a month	75.5	77.0
	43. Child has three or more books of his/her own	84.5	17.3
	Average	81.9	47.1

Source: Bradley et al. [42], own survey data.
